# Adult-onset Still’s disease during pregnancy: two case reports and a comprehensive literature review

**DOI:** 10.3389/fmed.2025.1588300

**Published:** 2025-07-09

**Authors:** Wenchao Xu, Shuang Huang, Juanli Li, Ting Li, Yu Lu, Liu Yang, Jianyu Zhang, Xue Li, Jian Chen

**Affiliations:** ^1^Department of Rheumatology and Immunology, The Third Affiliated Hospital, Guangzhou Medical University, Guangzhou, China; ^2^Guangdong Provincial Key Laboratory of Major Obstetric Diseases, Guangzhou, China; ^3^Guangdong Provincial Clinical Research Center for Obstetrics and Gynecology, Guangzhou, China

**Keywords:** pregnancy outcomes, Adult-onset Still’s disease, AOSD, safe medication use, inflammatory disorder

## Abstract

**Background:**

Adult-onset Still’s disease (AOSD) is a rare systemic inflammatory disorder marked by recurrent fever, rash, arthritis, and multi-organ involvement. Its occurrence with pregnancy complicates diagnosis and management.

**Objectives:**

To present the diagnosis and treatment process of two pregnant patients with newly diagnosed AOSD.

**Cases:**

Two cases of AOSD were initially diagnosed during pregnancy. Case one involved a pregnant woman at 16 + 2 weeks of gestation with recurrent fevers, rash, and myalgia. She responded well to treatment with methylprednisolone and cyclosporine and subsequently had a normal vaginal delivery. Case two involved a pregnant woman at 30 + 6 weeks of gestation who presented with systemic joint pain and fever. After being diagnosed with AOSD, she underwent a cesarean section. Treatment included methylprednisolone, cyclosporine, and addition of methotrexate postpartum.

**Conclusion:**

Adult-onset Still’s disease can be triggered by pregnancy, requiring a multidisciplinary approach for optimal management and fetal outcomes.

## 1 Introduction

Adult-onset Still’s disease (AOSD) is a rare inflammatory disorder characterized by fever, rash, arthritis, and multi-organ involvement. While conventional treatments with non-steroidal anti-inflammatory drugs (NSAIDs), corticosteroids, and DMARDs are effective in managing about 60% of cases (1), refractory cases often require biologics, though prospective trials and large-scale studies are scarce (2). The complexity of disease increases during pregnancy. This article reviews two cases of AOSD during pregnancy, focusing their clinical presentations, imaging, diagnosis, treatment and prognosis. Both patients showed improvement, were discharged, and had healthy pregnancies. This report aims to provides valuable insights for diagnosis and safe management of AOSD during pregnancy.

## 2 Case presentation 1

**FIGURE 1:**
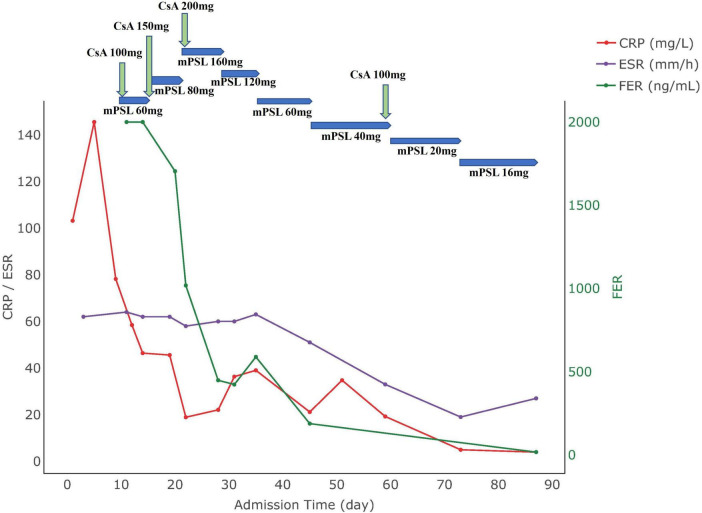
Medication use and biomarker trends in Case 1. CRP, C-reactive protein; ESR, erythrocyte sedimentation rate; FER, ferritin; CsA, cyclosporine A; mPSL, methylprednisolone.

A 28 years-old female patient, 16 + 2 weeks -of gestation, who was admitted to the hospital with a 1 week history of fever, rash, and myalgia. She experienced a peak temperature of 39°C. Dermatological examination revealed a rash characterized as flat clusters that faded when pressed, distributed across her head, face, neck, and limbs. Laboratory tests showed the following: white blood cell count (WBC):16.82 × 10^∧^9/L; neutrophil percentage (NE%): 93.7%; procalcitonin (PCT): 0.248 ng/ml; hemoglobin: 108 g/L, platelets: 159 × 10^∧^9/L, aminotransferase (ALT):11.7 U/L, aspartate transaminase (AST): 26.2 U/L, C-reactive protein (CRP) level 103.18 mg/L, erythrocyte sedimentation rate (ESR): 62 mm/h. Notably, autoantibodies were negative and serum ferritin was significantly elevated at over 2,000 μg/L. Bone marrow showed no abnormalities. CT scans identified localized fibroplasia in the lung and mild bilateral pleural thickening. Despite initial antimicrobial therapy, the patient experienced persistent fevers and elevated inflammatory markers after 5 days, indicating a lack of response to the treatment. Following a multidisciplinary consultation, the patient was diagnosed with AOSD and received intravenous methylprednisolone 60 mg daily and oral cyclosporine 50 mg twice daily. Although there was improvement in her rash, recurrent fevers persisted. Consequently, the regimen was adjusted to intravenous methylprednisolone 80 mg twice daily and cyclosporine 100 mg twice daily. Subsequent assessments showed improvements: WBC: 13.87 × 10^∧^9/L, NE%: 86.3%, PCT: 0.082 ng/ml, Hemoglobin: 108 g/L, Platelet count: 226 × 10^∧^9/L, ALT: 22.2 U/L, AST: 25.2 U/L, Serum Ferritin: 589.06 μg/L, ESR:63 mm/h, CRP:39.03 mg/L. Upon clinical stabilization, the patient was discharged with a tapering regimen of methylprednisolone and cyclosporine. During the later stage of pregnancy, the regimen was adjusted to steroids 8 mg once daily and cyclosporine 25 mg thrice daily. Subsequently, clinical markers normalized and her condition remained stable. Four months post-discharge, she delivered a healthy male infant vaginally at a local facility. The changes in the patient’s clinical parameters are illustrated in Figure 1, with the day of hospitalization marked as day 0.

## 3 Case presentation 2

A 25 years-old patient, 30 + 6 weeks of gestation, was admitted to hospital with generalized joint pain for 7 days, cough, sputum and fever for 3 days. On admission, she recorded a temperature of 38.8°C, with skin appearing normal in color and no rash. Laboratory tests indicated the following: WBC: 26.52 × 10^∧^9/L; NE%:92%; PCT: 1.02 ng/ml; hemoglobin: 89 g/L, platelets: 357 × 10^∧^9/L, ALT: 5.9 U/L, AST: 15 U/L, CRP: 196.84 mg/L. The CT showed pneumonia, moderate pleural effusion, small pericardial effusion. Ultrasound findings suggested fetal growth retardation. Despite treatment for infection, the patient’s condition persisted with recurrent fevers and new onset of a mild, transient rash. Symptomatology included severe chest pain, joint pain, and sore throat, which abated with the resolution of fever. Despite repeated episodes of high fever, systemic joint pain, and elevated levels of white blood cells, neutrophils, and liver enzymes, we were able to exclude infections, tumors, and other immunological disorders. Based on the fulfillment of the Yamaguchi criteria (3), she was diagnosed with AOSD. The therapeutic strategy included 60 mg of methylprednisolone daily along with 50 mg of cyclosporine twice daily. This regimen led to improvements in joint pain and sore throat, although her fever continued. Further tests revealed the following: WBC 22.21 × 10^∧^9/L, NE%: 92.2%, hemoglobin levels at 88 g/L, platelet: 374 × 10^∧^9/L, ESR: 69 mm/h, CRP: 91.6 mg/L. Given the ultrasound evidence of continued fetal growth restriction, a decision was made to deliver via cesarean section at 34 weeks, resulting in the birth of a newborn weighing 1,400 g and measuring 39 cm in length. Following surgery, the patient commenced treatment with 10 mg of methotrexate weekly. This regimen moderated fever peaks, but intermittent fevers persisted. A follow-up examination after the cesarean section revealed the following: white blood cells at 29.43 × 10^∧^9/L, NE:88.2%, hemoglobin: 91 g/L, platelets: 557 × 10^∧^9/L, serum ferritin: 421.

**FIGURE 2:**
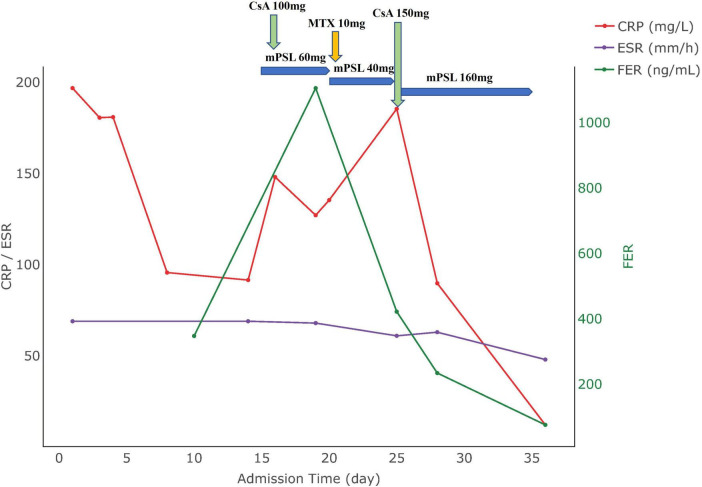
Medication use and biomarker trends in Case 2. CRP, C-reactive protein; ESR, erythrocyte sedimentation rate; FER, ferritin; CsA, cyclosporine A; mPSL, methylprednisolone.

62 μg/L, ESR:61 mm/h, CRP:185.48 mg/L, prompting an adjustment in her regimen to include 75 mg of cyclosporine twice daily. Subsequent tests showed normalized body temperature and substantial resolution of pulmonary inflammation as evidenced by chest CT. The patient’s condition stabilized with improved hematological markers, allowing for discharge. Post-discharge follow-up indicated the infant was healthy, and the patient had been treated with adalimumab at an external hospital, but symptoms still occasionally recurred mildly. The changes in the patient’s clinical parameters are illustrated in Figure 2, with the date of hospital admission noted as day 0.

## 4 Discussion

Adult-onset Still’s disease is a systemic inflammatory disorder with unknown etiology, characterized by a constellation of symptoms including high fever, lymphadenopathy, hepatospleno-megaly, methemoglobinemia, and leukocytosis (4). Globally, the incidence of AOSD is estimated to range between 1.6 and 4.0 cases per million individuals annually (4). The diagnosis of AOSD is primarily based on clinical manifestations, alongside laboratory indicators such as elevated white blood cell counts and serum ferritin levels, while excluding infections, tumors, and other rheumatologic conditions. The occurrence of pregnancy introduces additional diagnostic challenges, complicating the clinical presentations and necessitating a careful consideration of both maternal and fetal health. Currently, no universally recognized diagnostic standard for AOSD, particularly in pregnant patients. Among various diagnostic criteria, the Yamaguchi criteria are noted for the high sensitivity (5). In the two cases discussed in this article, after exclusion of infections and tumors, a definitive diagnosis of AOSD was established based on the Yamaguchi criteria.

### 4.1 The relationship between AOSD and pregnancy

#### 4.1.1 The impact of pregnancy on AOSD

The potential influence of pregnancy on the induction of AOSD was first reported by Green et al. (6). However, the underlying biological mechanisms remain unclear. Research indicated that estrogens can activate macrophages, leading to increased production of tumor necrosis factor (TNF-α), IL-6, and IL-1 (7). These cytokines play critical roles in inflammatory processes, and estrogens can further enhance the expression of IL-1 mRNA in monocytes while also augmenting several endothelial cell functions, including adhesion to matrix proteins, migration, differentiation, and inflammation (8). Additionally, recent studies indicated a correlation between elevated IL-18 levels during pregnancy and the pathogenesis of AOSD (9, 10). Notably, pregnancy may act as a trigger for AOSD recurrence, particularly during early to mid-pregnancy and the postpartum period, even in patients in clinical remission (11). Wang et al. (12) highlighted that pregnancy could increase the risk of AOSD onset, which may manifest in various clinical patterns: monocyclic, polycyclic, and chronic. Pregnancy-associated AOSD tends to follow monocyclic or polycyclic patterns, with chronic arthritis being relatively uncommon (12, 13). In our study, the initial symptoms of AOSD in both patients were observed during the mid- and late-pregnancy stages, with the first patient presenting a monophasic pattern and the second patient experienced recurrent symptoms indicative of a polycyclic pattern.

#### 4.1.2 The impact of AOSD on pregnancy outcomes

Limited research indicates that the presence of AOSD in pregnant women has been associated with an increased risk of adverse pregnancy outcomes. Leo et al. reported a case-based review, which analyzed 19 cases of AOSD manifested during pregnancy reported obstetrical complications in nearly 50% of these cases, including prematurity (10/20), pre-term premature rupture of membranes (3/20), intrauterine growth restriction (3/20), oligohydramnios (2/20), or neonatal death (1/20) (12). Therefore, these findings suggest that fetal growth restriction may be directly attributable to AOSD, as observed in the second case of our study. Furthermore, a Chinese cohort study found that pregnancies following an AOSD diagnosis had a higher rate of spontaneous abortion (18.8%) compared to those occurring before diagnosis (0.6%). This underlies the increased risk of spontaneous abortion in pregnancies subsequent to an AOSD diagnosis (12).

### 4.2 Safe medication use in pregnancy with AOSD

While standardized treatment guidelines for AOSD remain under establishment, the current pharmacological treatment generally includes non-steroidal anti-inflammatory drugs (NSAIDs), corticosteroids, disease-modifying anti-rheumatic drugs (DMARDs) and biologic agents. However, studies have shown that NSAIDs often fail to control AOSD symptoms and are associated with a high risk of adverse events (1, 5, 14). Corticosteroids, despite potential complications such as gestational diabetes, arterial hypertension, and premature rupture of membranes, are considered the primary therapy for AOSD (15, 16). Among DMARDs, cyclosporine is recommended by EULAR guidelines as a viable antirheumatic option before, during, and post-pregnancy (17), and cyclosporine is particularly effective in achieving remission rates among steroid-resistant patients on single-agent DMARD therapy (18). In our clinical experience, we achieved positive results using steroids combined with cyclosporine for the first patient. However, for the second patient, who showed no response to cyclosporine alone, a combination of methotrexate and cyclosporine was effective postpartum.

The lack of clinical response with the first-line corticosteroids and second-line DMARDs may identify refractory AOSD patients (19). We believe that the patient in Case 2 can be considered a refractory case. Observational studies report that 17%–32% of AOSD patients may develop resistance to first-line corticosteroids and second line DMARDs (16, 17, 20). In such cases, biologics have been shown to frequently lead to clinical improvement and complete remission, as demonstrated by a meta-analysis of observational studies (21). Systemic inflammation in AOSD is primarily driven by pro-inflammatory cytokines such as IL-1, IL-6, IL-18, IL-17, and TNF-α, which are associated with disease activity (22). The administration of IL-1 and IL-6 inhibitors in pregnancy-associated AOSD has been reported without adverse effects (23–26). The treatment with TNF-α inhibitors has been associated with oligohydramnios and fetal growth restriction, though it is unclear whether these outcomes are attributed to the medication or the disease itself (26). For non-pregnant AOSD patients, IL-1 inhibitors are recommended as first-line biologics agents, while EULAR notes that TNF-α antagonists are the most frequently used in pregnant patients with autoimmune diseases (17).

## 5 Conclusion

The clinical manifestations of AOSD often mimic those of other inflammatory and infectious diseases, resulting in delayed diagnosis. This delay is particularly critical during pregnancy, as untreated AOSD can significantly elevate mortality rates for both mother and fetus. Therefore, prompt and accurate diagnosis is essential, especially for patients experiencing their first AOSD episode during pregnancy. Currently, there is no consensus on treatment protocols for AOSD in pregnant patients. For refractory AOSD patients during pregnancy, the administration of biologics, alongside cyclosporine, presents a viable treatment option. Considering the complex interplay between AOSD and pregnancy, it is recommended that patient management be approached through a multidisciplinary strategy involving both rheumatologists and obstetricians, particularly during the perinatal period. This collaborative approach is crucial for optimizing both maternal and fetal outcomes.

## Data Availability

The original contributions presented in this study are included in this article/supplementary material, further inquiries can be directed to the corresponding authors.
